# Genetic analysis of *ERBB4* gene in Chinese patients with amyotrophic lateral sclerosis: a single-center study and systematic review of published literature

**DOI:** 10.3389/fnagi.2025.1584541

**Published:** 2025-05-21

**Authors:** Dongchao Shen, Xunzhe Yang, Di He, Kang Zhang, Shuangwu Liu, Xiaohan Sun, Jinyue Li, Zhengyi Cai, Mingsheng Liu, Xue Zhang, Qing Liu, Liying Cui

**Affiliations:** ^1^Department of Neurology, Peking Union Medical College Hospital, Beijing, China; ^2^McKusick-Zhang Center for Genetic Medicine, Chinese Academy of Medical Sciences and Peking Union Medical College, Beijing, China; ^3^Neuroscience Center, Chinese Academy of Medical Sciences and Peking Union Medical College, Beijing, China

**Keywords:** ERBB4 variants, amyotrophic lateral sclerosis, genetic screening, ethnic differences, disease modifiers

## Abstract

**Background:**

Rare *ERBB4* variants have been implicated in amyotrophic lateral sclerosis (ALS), but their prevalence and clinical significance remain poorly understood, particularly across different ethnic populations.

**Methods:**

We performed genetic screening of *ERBB4* in 1627 Chinese ALS patients using whole-exome sequencing. A systematic review and meta-analysis of the published literature were conducted to evaluate the global frequency of *ERBB4* variants and their clinical correlations.

**Results:**

We identified 14 missense variants and 6 splice region variants in 23 unrelated patients, with four variants classified as damaging (p.R782P, p.M799T, p.R847C, and p.S997R). The splice variant c.1490-3C > T, associated with a 50% reduction in *ERBB4* mRNA expression, was maternally inherited by a male ALS patient, while its presence in his asymptomatic mother suggests the involvement of potential genetic modifiers. *ERBB4* variant carriers demonstrated earlier disease onset compared to non-carriers (46.9 ± 10.3 vs. 52.6 ± 11.2 years; *p* = 0.015), though survival duration remained comparable. Meta-analysis revealed a pooled *ERBB4* variant frequency of 0.83% (95% CI, 0.56–1.10%) in ALS patients globally, with notable ethnic differences (1.36% in Chinese, 0.66% in European, and 1.44% in American populations).

**Conclusion:**

Our findings establish the prevalence of *ERBB4* variants in ALS across different populations and suggest their potential role as disease modifiers, particularly affecting the age of onset. The ethnic variation in mutation frequency highlights the importance of population-specific genetic screening strategies in ALS.

## Introduction

Amyotrophic lateral sclerosis (ALS) is a fatal neurodegenerative disorder that affects both upper and lower motor neurons, with a global annual incidence of 1.68 per 100,000 individuals (Feldman et al., 2022). The causes of ALS remain largely elusive. While over 50 genes have been implicated in ALS pathogenesis, currently identified mutations explain ~47.7% of familial (fALS) and 5.2% of sporadic cases (sALS) (Zou et al., 2017). Genetic factors play a crucial role in the pathogenesis of ALS, and there is significant genetic diversity among ethnic groups impacted by the disease. Our prior whole-exome sequencing study revealed distinct mutation spectra between Chinese and Caucasian cohorts, particularly *C9orf72* repeat expansions (0.12% vs. 5.21%, respectively) (Yang et al., 2022; Van Daele et al., 2023).

The *ERBB4* mutation, first identified in a family of ALS patients in 2013, was recognized as a new pathogenic gene for ALS19 (Takahashi et al., 2013). It encodes a type I transmembrane receptor tyrosine kinase that dimerizes upon binding neuregulin (NRG) ligands, initiating downstream PI3K-AKT and MAPK signaling cascades critical for neuronal survival (Mei and Nave, 2014). Reduced ERBB4 immunoreactivity in spinal cord tissues has been shown to correlate with the severity of motor neuron degeneration and the mislocalization of TDP-43 in postmortem studies of ALS patients (Takahashi et al., 2019). Genome-wide association studies have identified a significant association between a polymorphism in *ERBB4* and the pathogenesis of sALS, particularly within the nervous system developmental pathway (Deng et al., 2017). Additionally, emerging evidence links specific *ERBB4* variants to the ALS-FTD continuum, demonstrating that the c.2136 T > G (p.I712M) mutation can reduce autophosphorylation of the ERBB4 protein following NRG1 stimulation *in vitro* (Sun et al., 2020). NRG1-III exhibits significant benefits in ALS by preserving motor neuron function and survival, particularly by maintaining neuromuscular junctions and reducing neuroinflammation in SOD1G93A mice (Lasiene et al., 2016; Modol-Caballero et al., 2020), highlighting the therapeutic potential of targeting the NRG1-ERBB4 axis in ALS. However, the prevalence and impact of *ERBB4* variants on phenotype in the ALS population remain poorly understood. In this study, we aim to investigate the frequency of *ERBB4* variants in a large Chinese ALS cohort, integrate data from previously reported *ERBB4* variants, and explore genotype–phenotype correlations.

## Methods

### Study population

This study enrolled 1,627 patients diagnosed with definite, probable, or laboratory-supported probable ALS according to the revised El Escorial criteria (Brooks et al., 2000) at the neurology department of Peking Union Medical College Hospital (PUMCH) from January 2014 to December 2022. The diagnosis of ALS-FTD was established based on the Strong criteria (Strong et al., 2017). This cohort included 89 unrelated fALS probands, 1,583 sALS patients and 52 ALS-FTD cases. The study received approval from the Ethics Review Board of PUMCH (approval number I-23PJ1218), and all participants provided informed written consent.

### Genetic testing and bioinformatics analysis

According to standard protocols, patient and control genomic DNA was extracted from peripheral blood leucocytes (Blood DNA Kit V2, CW2553). We conducted whole-exome sequencing (WES) to analyze *ERBB4* and other known ALS-related genes, such as *SOD1*, *FUS*, *ANXA11*, among others (Shen et al., 2024). The HiSeq2000 system (Illumina, San Diego, California, USA) was utilized to generate paired-end 200 bp reads. We retained variants with a read depth of ≥10 and a genotype quality of ≥20. The average sequencing depth exceeded 100X, with coverage surpassing 98.6%. Alignment to the human reference genome (UCSC hg19) was performed using the Burrows-Wheeler Aligner, followed by reformatting with SAM tools. To exclude common single nucleotide polymorphisms (SNPs), variant frequencies were assessed in gnomAD (East Asian, EA) and ChinaMAP.^1^ ChinaMAP is constructed from cohort studies encompassing a broad range of regions and ethnicities across China. In its beta version (2020–03), the database reports allele frequencies for genetic variants observed in 10,588 Chinese individuals. These individuals were chosen at random from 27 provinces and represent eight distinct ethnic groups, including Han, Hui, Manchu, Miao, Mongolian, Yi, Tibetan, and Zhuang, without any selection bias or filtering (Cao et al., 2020). As a result, the ChinaMAP resource serves as a comprehensive and representative reference for the genetic background of the general Chinese population. For further analysis, we selected only non-synonymous, splicing, and frameshift variants with a minor allele frequency (MAF) below 0.5% across all population databases. Variant annotation and filtering were conducted using SIFT, PolyPhen2, CADD and Mutation Taster. The functional effects of the splicing variants were predicted using Human Splicing Finder,^2^ Splice AI.^3^ RDDC Splicing^4^ and var. SEAK.^5^ Additionally, fragment-length and repeat-primed Polymerase Chain Reaction were employed to identify duplications or deletions in *ATXN2* and *C9ORF72* among ALS-FTD patients. Interpretation of causative mutations was primarily based on Clinvar. database and the guidelines of the American College of Medical Genetics and Genomics (ACMG) (Richards et al., 2015). The relative mRNA expression of *ERBB4* was determined for patients carrying c.1490-3C > T variant using reverse transcription quantitative polymerase chain reaction (RT-qPCR) with the following primers: forward 5’-GAGTACTCTATAGTGGCCTG-3′, reverse 5’-TTTGCCCCCTGTAAGCCATCT −3′.

### Systematic review of the literature and statistical analysis

We conducted a systematic literature review of ALS patients with *ERBB4* variants by searching MEDLINE-PubMed and Embase and Web of Science for studies published between November 2013 and December 2024 using combinations of the following keywords: “amyotrophic lateral sclerosis,” “*ERBB4*,” and “genetic.” We did not contact the authors of the included studies or seek unpublished data. Two authors independently extracted the following information: the number of patients in each study, age at disease onset, gender, variant details, site of onset, and family history.

Meta-analysis was carried out using OpenMeta software.^6^ The effect sizes and 95% confidence intervals (CIs) for each study were calculated. Statistical heterogeneity was assessed using Cochrane’s Q test (*p* < 0.10) and I^2^ statistics. Studies with I^2^ < 50% were deemed homogeneous, and a fixed-effects model was applied to combine variant frequencies. Conversely, I^2^ ≥ 50% or p < 0.10 indicated substantial heterogeneity, warranting the use of a random-effects model. Leave-one-out sensitivity analyses were performed to evaluate the robustness of results by excluding one study at a time and recalculating the stability of the remaining studies. Clinical data processing and analysis were conducted using SPSS statistical software version 22.0. For continuous data that followed a normal distribution, the mean ± standard deviation was used for description, and comparisons between groups were made using independent sample t-tests. For continuous data that did not follow a normal distribution, medians and interquartile ranges (IQRs) were used, and the Mann–Whitney U test was applied for analysis. All statistical calculations were considered significant at a *p*-value of less than 0.05.

## Results

### Pathogenicity analysis of ERBB4 rare variants in our cohort

The detailed clinical and genetic characteristics of the entire cohort have been published elsewhere (Shen et al., 2024). Genetic screening of the *ERBB4* gene in a total of 1,627 ALS patients revealed 14 missense variants (Table 1) and 6 splice region variants (Table 2) in 23 unrelated ALS patients. None of the 6 splice region variants were classified as damaging. Based on in-silico predictions, four heterozygous variants including c.2345G > C (p.R782P), c.2396 T > C (p.M799T), c.2539C > T (p.R847C), and c.2989A > C (p.S997R) were classified as damaging. The variants are all located within the catalytic domain of the protein tyrosine kinase, with one or more neighboring residues harboring known pathogenic mutations (Takahashi et al., 2013; Chen et al., 2020; Borg et al., 2021; Liu et al., 2021; Farrugia Wismayer et al., 2023). These predicted damaging variants in the catalytic domain may interfere with the kinase function of ERBB4.

**Table 1:** Prediction of pathogenicity in *ERBB4 missense* variants identified in this study.

Chr:Position GRCh37	Variants	dbSNP	Frequency in patient allele	GnomAD_EA	China map	SIFT	Poly-Phen2	CADD	Mutation taster	ClinVar
2:212615376	c.610C > G (p.H204D)	Novel	1/1627	-	-	Tolerable	Benign	Tolerable	Disease_causing	Not Reported
2:212589887	c.655G > A (p.G219S)	rs757597004	1/1627	1/18392	1/21176	Damaging	Probably_damaging	Damaging	Disease_causing	Uncertain significance
2:212578283	c.974C > T (p.P325L)	rs1162491783	1/1627	2/19952	-	Tolerable	Probably_damaging	Damaging	Disease_causing	Not Reported
2:212495294	c.1972A > T (p.I658F)	rs190654033	2/1627	68/19952	123/21176	Tolerable	Probably_damaging	Damaging	Disease_causing	Likely benign
**2:212426770**	**c.2345G > C (p.R782P)**	**rs747423735**	**1/1627**	**-**	**-**	**Damaging**	**Probably_damaging**	**Damaging**	**Disease_causing**	**Not Reported**
**2:212426719**	**c.2396 T > C (p.M799T)**	**rs367778613**	**1/1627**	**0/18392**	**-**	**Damaging**	**Probably_damaging**	**Damaging**	**Disease_causing**	**Not Reported**
**2:212295774**	**c.2539C > T (p.R847C)**	**rs754127388**	**1/1627**	**0/18394**	**-**	**Damaging**	**Probably_damaging**	**Damaging**	**Disease_causing**	**Not Reported**
2:212288916	c.2830A > G (p.I944V)	rs1435419609	1/1627	3/18392	-	Tolerable	Benign	Tolerable	Disease_causing	Not Reported
2:212286761	c.2935C > G (p.R979G)	rs574197848	1/1627	4/18390	8/21176	Damaging	Possibly_damaging	Damaging	Disease_causing	Not Reported
**2:212285312**	**c.2989A > C (p.S997R)**	**Novel**	**1/1627**	**-**	**-**	**Damaging**	**Possibly_damaging**	**Damaging**	**Disease_causing**	**Not Reported**
2:212285256	c.3045 T > G (p.D1015E)	Novel	1/1627	-	-	Tolerable	Benign	Tolerable	Disease_causing	Not Reported
2:212251688	c.3371G > A (p.S1124N)	rs1356804335	1/1627	0/18394	-	Tolerable	Benign	Tolerable	Disease_causing	Not Reported
2:212251595	c.3464G > A (p.R1155Q)	rs1044752647	1/1627	0/19952	-	Tolerable	Benign	Damaging	Polymorphism	Not Reported
2:212248746	c.3521G > A (p.R1174Q)	rs762661533	1/1627	2/18392	2/21176	Tolerable	Possibly_damaging	Damaging	Disease_causing	Not Reported

The 4 variants in bold were likely to be damaging.

**Table 2:** Prediction of pathogenicity in *ERBB4 splicing* variants identified in this study.

Chr:Position (GRCh37)	Variant	dbSNP	Frequency in patient allele	GnomAD_EA	China map	Human splicing finder	Splice AI ∆ score*	RDDC splicing alteration	var. SEAK	ClinVar
2:213403165	c.82 + 8C > T	rs200527371	1/1627	14/19922	26/21176	No significant impact	0.04	No	No splicing effect	Likely benign
2:212578379	c.884-7_884-6insTT	rs67894136	1/1627	7/11554	-	No significant impact	0.00	No	No splicing effect	Not Reported
2:212578380	c.884-7 T > A	rs74499552	1/1627	0/11618	-	No significant impact	0.00	No	No splicing effect	Not Reported
2:212543912	c.1490-3C > T	rs1411700213	3/1627	1/18384	2/21176	No significant impact	0.07	No	Likely no splicing effect	Not Reported
2:212543914	c.1490-5C > G	rs1484990292	1/1627	-	1/21176	No significant impact	0.10	No	Likely no splicing effect	Not Reported
2:212252722	c.3136-5A > G	rs188179744	1/1627	10/19946	35/21176	Potential alteration	0.05	No	No splicing effect	Likely benign

^*^Delta scores range from 0 to 1 and can be interpreted as the probability that the variant affects splicing at any position within a window around it (+/− 500 bp by default).

Notably, a 38-year-old male patient carried both the *OPTN* c.1634G > A (uncertain significance) and *ERBB4* c.1490-3C > T variants (Appendix Figure 1), and family verification revealed that both variants were maternally inherited. RT-qPCR revealed approximately a 50% reduction in *ERBB4* mRNA expression in the peripheral blood of the male patient compared to healthy controls (Appendix Figure 2). This suggests that the c.1490-3C > T variant leads to the degradation of *ERBB4*, consistent with the loss-of-function mutation mechanism associated with this gene. Interestingly, his asymptomatic mother, who also carries the same variant, exhibited no ALS-related symptoms or abnormalities on electromyography. The underlying mechanism that accounts for the phenotypic discrepancy within the family warrants further investigation.

### Clinical features of ALS patients with ERBB4 variants in our cohort

The genetic and clinical features of the 23 patients with *ERBB4* variants are summarized in Table 3. Among 23 ALS patients with rare *ERBB4* variants, the average onset age was 46.9 ± 10.3 years, significantly lower than that of the overall ALS cohort (Shen et al., 2024) (52.6 ± 11.2; T = 2.6, *p* = 0.015). The ratio of males to females was 14:9, the ratio of limb-onset to bulbar-onset cases was 18:5, and two patients had a family history of ALS. None of the patients exhibited cognitive impairment. Survival data were available for 14 patients, with a median survival period of 35 months (IQR 33–40), and no significant difference was observed compared to the overall ALS cohort [35 months (IQR 21–44)].

**Table 3:** Clinical characteristics of patients with *ERBB4 variants* in our cohort.

Variant	Gender	Age of onset (years)	Site of onset	Survival time (Months)	Family history	Multiple ALS-related rare variants
c.82 + 8C > T	Male	42	Limb	NA	No	
c.610C > G (p.H204D)	Male	47	Limb	NA	No	
c.655G > A (p.G219S)	Male	51	Limb	37	No	
c.884-7_884-6insTT	Male	49	Bulbar	40	No	
c.884-7 T > A	Male	57	Limb	NA	No	
c.974C > T (p.P325L)	Female	68	Limb	32	No	
c.1490-3C > T	Male	51	Bulbar	33	No	*HNRNPA*1 c.847G > A p.G283R
	Female	27	Limb	35	No	
	Male	38	Limb	42#	No	*OPTN* c.1634G > A p.R545Q
c.1490-5C > G	Male	49	Limb	39	No	
c.1972A > T (p.I658F)	Female	43	Limb	34	No	
	Male	44	Bulbar	83	No	*SPG11* c.581C > T p.P194L
**c.2345G > C (p.R782P)**	**Female**	**43**	**Limb**	**59**	**No**	
**c.2396 T > C (p.M799T)**	**Female**	**38**	**Limb**	**61**	**No**	
**c.2539C > T (p.R847C)**	**Male**	**49**	**Limb**	**28**	**No**	
c.2830A > G (p.I944V)	Female	55	Bulbar	30	No	
c.2935C > G (p.R979G)	Male	53	Limb	NA	No	
**c.2989A > C (p.S997R)**	**Male**	**51**	**Limb**	**NA**	**No**	
c.3045 T > G (p.D1015E)	Female	46	Bulbar	38#	No	
c.3136-5A > G	Female	18	Limb	35	No	
c.3371G > A (p.S1124N)	Female	59	Limb	34	No	*SOD1* c.37G > C p.G13R*
c.3464G > A (p.R1155Q)	Male	52	Limb	52#	Yes	
c.3521G > A (p.R1174Q)	Male	49	Limb	NA	Yes	

ALS, amyotrophic lateral sclerosis; NA, not available, # Alive. *Known pathologic mutation. The 4 variants in bold were likely to be damaging.

### Genotype–phenotype association in ALS patients with *ERBB4* variants

**Figure 1:**
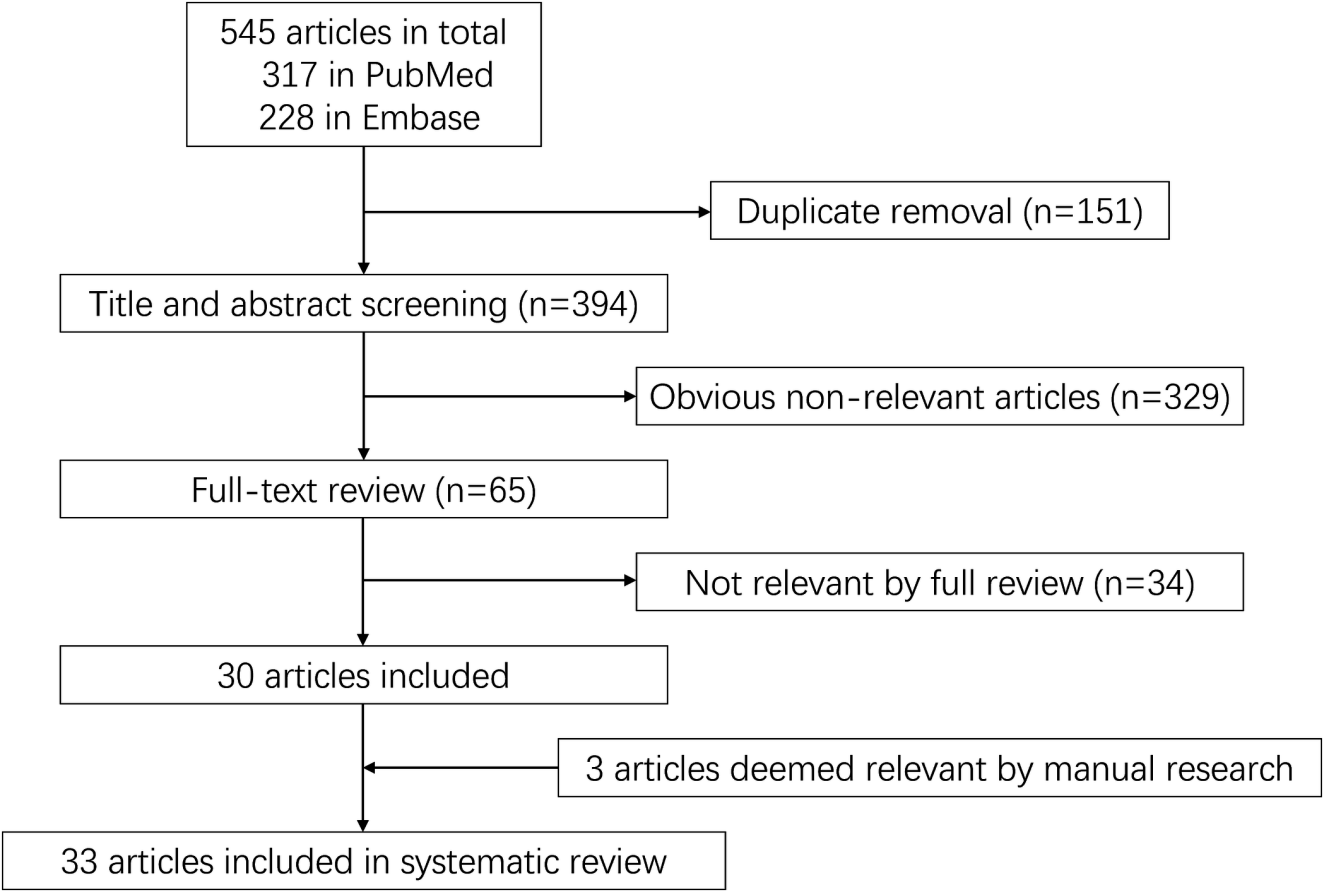
The flow chart of the literature search in the systematic review.

**Figure 2:**
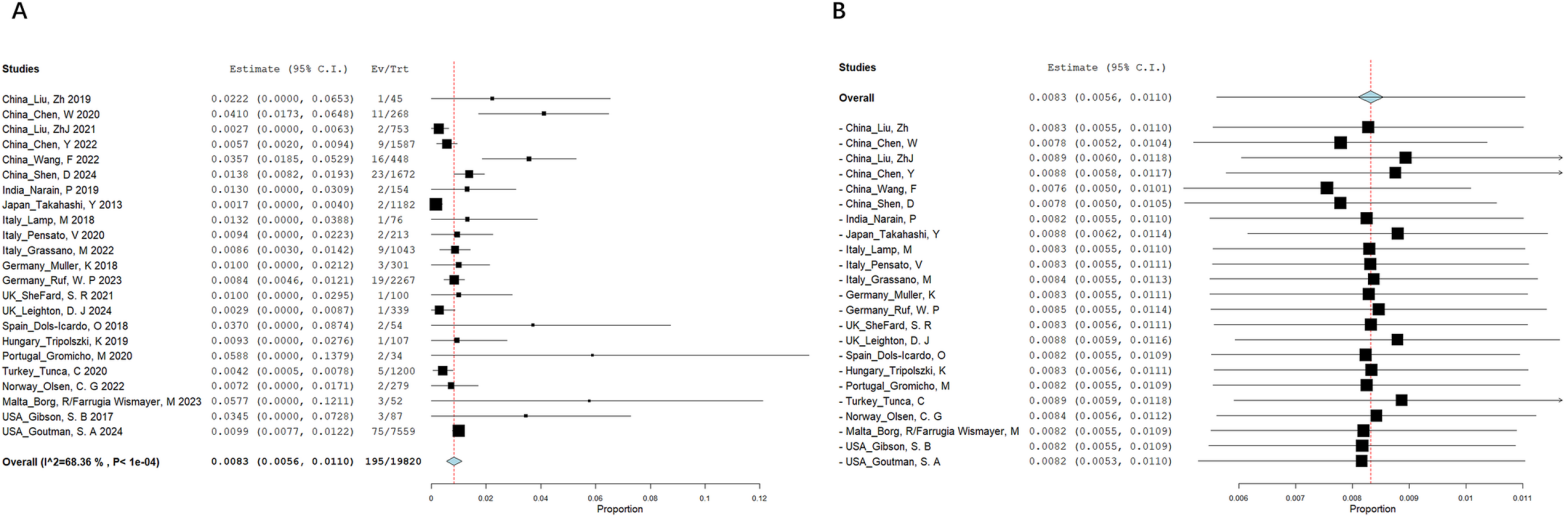
Meta-analysis of *ERBB4* variant frequency in ALS patients. **(A)** Forest plot. **(B)** Leave-one-out analysis.

**Figure 3:**
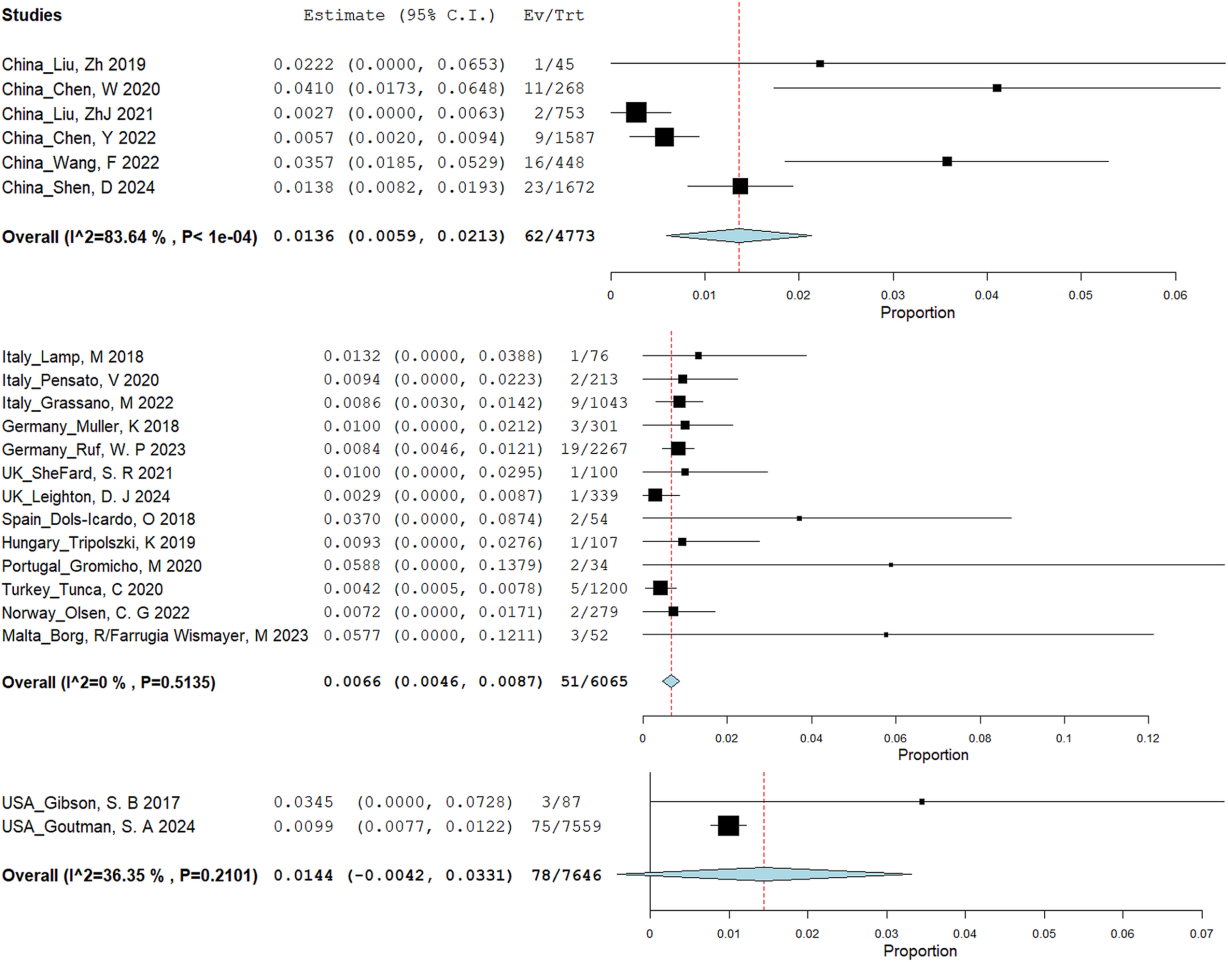
Forest plot of studies reporting ERBB4 variants in ALS in Chinese, European and American.

A total of 545 articles were identified in 2 databases: 317 in PubMed and 228 in Embase. After removing duplicate and unrelated articles, 32 articles remained. Additionally, 3 more relevant articles were identified through a search of the Human Gene Mutation Database (Professional 2024.04) (Figure 1). A total of 185 rare variants of ERBB4 associated with ALS were reported among 229 individuals in 33 articles (Takahashi et al., 2013; Gibson et al., 2017; Brenner et al., 2018; Dols-Icardo et al., 2018; Lamp et al., 2018; Müller et al., 2018; Narain et al., 2018; Capalbo et al., 2019; Liu et al., 2019; Narain et al., 2019; Tripolszki et al., 2019; Chen et al., 2020; Gromicho et al., 2020; Pensato et al., 2020; Sun et al., 2020; Tunca et al., 2020; Borg et al., 2021; Liu et al., 2021; Shepheard et al., 2021; Sun et al., 2021; Chen et al., 2022; Grassano et al., 2022; Olsen et al., 2022; Wang et al., 2022; Yilmaz et al., 2022; Farrugia Wismayer et al., 2023; Gorukmez et al., 2023; Ruf et al., 2023; Van Daele et al., 2023; Zhang et al., 2023; Goutman et al., 2024; Kwon et al., 2024; Leighton et al., 2024) and our study (Hu et al., 2024; Shen et al., 2024) (Appendix Table 1). Of the 185 rare variants reported, five—c.655G > A (p.G219S), c.974C > T (p.P325L), c.1972A > T (p.I658F), c.3136-5A > G, and c.3464G > A (p.R1155Q)—were identified in our study population. Among the 185 rare variants of *ERBB4*, a total of 33 were classified in the original article as pathogenic, likely pathogenic, or damaging. In these 35 studies, one conducted a retrospective analysis to determine the diagnostic yield of clinical exome sequencing in 1589 patients with a wide phenotypic spectrum, identifying a 44-year-old individual with a loss of ability to walk who carried a *de novo ERBB4* c.919G > T (p.A307S) variant (Gorukmez et al., 2023). Another study performed exome sequencing on either gamete donors or infertile patients undergoing *in vitro* fertilization treatment without any known family history of inheritable genetic conditions, identifying an asymptomatic individual carrying the *ERBB4* c.1630C > T (p.R544W) variant (Capalbo et al., 2019). However, due to the unclear diagnoses of these two cases, they were not included in the final clinical analysis. Excluding four case reports (Sun et al., 2020; Yilmaz et al., 2022; Zhang et al., 2023; Kwon et al., 2024), the pooled frequency of *ERBB4* variants in patients with ALS was 0.83% (95% CI: 0.56–1.1%). Significant heterogeneity was observed (random effects model, I^2^ = 68.4%, *p* < 0.001) (Figure 2A), primarily driven by studies conducted in China (Figure 2B). After pooling, the frequencies of *ERBB4* variants were 1.36% in China, 0.66% in Europe, and 1.44% in the United States, respectively (Figure 3).

The variant c.1972A > T (p.I658F) is the most frequently occurring variant, with a total of 8 patients. The variants c.308G > A (p.R103H) and c.3446G > T (p.G1149V) each have 4 patients, while c.268G > T (p.A90S), c.1490-3C > T, c.1720C > A (p.P574T), c.3464G > A (p.R1155Q), and c.3823C > T (p.R1275W) each have 3 patients. Additionally, there are 23 other variants, each found in two ALS patients, and variant c.3335G > A (p.R1112H) was found in both an ALS patient and an FTD patient (Tábuas-Pereira et al., 2022; Goutman et al., 2024). Among the remaining 227 ALS patients, 5 patients were diagnosed with ALS-FTD (c.1122 T > G p.H374Q, c.1240 T > C p.F414L, c.1997 T > C p.I666T, c.2136 T > G p.I712M, c.2728A > G p.I910V), and 11 patients had multiple variants. In the limited clinical data obtained, the average onset age was 48.6 ± 10.7 years, the male-to-female ratio was 32:19, the limb-onset to bulbar-onset ratio was 42:11, and the ratio of fALS to sALS was 15:96. There was no significant difference in age of onset between the pathogenic variant group (n = 15) and the non-pathogenic variant group (*n* = 37) (51.9 ± 10.0 vs. 47.0 ± 10.8; T = 1.6, *p* = 0.115). Similarly, no significant difference was observed between patients with (*n* = 11) and without (*n* = 39) a family history (51.1 ± 11.3 vs. 47.2 ± 10.0; T = 1.1, *p* = 0.269). There is insufficient survival data to conduct a meaningful analysis.

In our study, we identified 4 patients with rare variants in both *ERBB4* and other ALS-related genes (Table 3). Combined with previously published studies, a total of 11 such patients have been reported (Appendix Table 2). Among these, 3 cases carried pathogenic SOD1 mutations (Grassano et al., 2022; Ruf et al., 2023; Shen et al., 2024), 1 case had a pathogenic C9orf72 expansion (Olsen et al., 2022), and the remaining carriers of multiple gene variants had overlapping genes of uncertain significance. One sALS case carried rare nonsynonymous variants in four known ALS genes, with up to three of these variants also detected in unaffected, older family members, suggesting that ALS may result from the additive or synergistic effects of low-impact mutations (Yilmaz et al., 2022). Chen et al. reported that patients with the *ERBB4* gene tended to have multiple variants, but the study did not include specific details (Chen et al., 2020). The clinical information available for the 11 patients is insufficient, leaving the impact of rare *ERBB4* variants on the exacerbation of the ALS phenotype uncertain.

## Discussion

Our analysis identified 23 rare *ERBB4* variants in 1,627 Chinese ALS patients, with a prevalence of 2.25% in fALS and 1.33% in sALS, of which 4 were classified as damaging. A meta-analysis integrating global cohorts revealed ethnic heterogeneity, with a lower mutation prevalence in European populations compared to their Chinese and American counterparts. Clinical correlations demonstrated an earlier symptom onset in variant carriers; however, definitive genotype–phenotype associations were lacking.

The pathogenicity of these four damaging variants is supported by multiple lines of evidence. Firstly, they are all situated within a well-defined functional domain known to lack benign variation (PM1). The catalytic domain of the protein tyrosine kinase (amino acids 710–1,012) is essential for ERBB4’s enzymatic activity, including NRG-1-induced autophosphorylation, which mediates downstream signaling crucial for motor neuron survival and function. Secondly, none of these variants have been reported in population databases such as gnomAD_EA or ChinaMAP (PM2). Furthermore, in silico prediction tools consistently identified these variants as disease-causing, and they exhibit strong evolutionary conservation across species (PP3). Lastly, the clinical phenotypes of the affected patients are highly consistent with the disease linked to ERBB4 variants (PP4). According to the ACMG variant classification criteria, these four variants are therefore classified as “likely pathogenic” (PM1 + PM2 + PP3 + PP4). The splice variant c.1490-3C > T is relatively frequent in our cohort, and RT-qPCR results indicate that a patient with this variant exhibits an approximately 50% reduction in peripheral blood *ERBB4* mRNA expression. The mother carrying the same variant shows no clinical or subclinical manifestations related to ALS, which does not rule out the possibility of her having protective or modifier genes. Otherwise, it may also be possible that ERBB4 does not cause ALS in a loss-of-function way.

The carrier rate of *ERBB4* variants exhibits considerable variability across ALS cohorts, ranging from 0.17 to 5.9%, with a pooled variant carrier rate estimated at 0.83% (95% CI: 0.56–1.1%). These discrepancies may be attributed to differences in patient sample sizes and the screening methodologies employed. Additionally, the observed variation among ethnic groups highlights potential genetic heterogeneity and population-specific contributions to ALS pathogenesis. The most frequently occurring variant c.1972A > T (p.I658F) is carried by individuals who are all Chinese, with a relatively high frequency both in gnomAD_EA (0.34%) and ChinaMAP (0.58%). It has also been detected in a Chinese male patient with isolated hypogonadotropic hypogonadism (Zhou et al., 2018) and is classified as likely benign in ClinVar. The second most frequent variants, c.308G > A (p.R103H) and c.3446G > T (p.G1149V), are classified as having conflicting interpretations of pathogenicity and uncertain significance in ClinVar, respectively.

Although definitive genotype–phenotype associations remain unclear, it is noteworthy that carriers of *ERBB4* variants tend to have a relatively younger age of onset, suggesting a potential role as an ALS age-of-onset modifier. This is further supported by findings showing that the mean age of onset in individuals with *ERBB4* gene insertions is approximately 1 year younger than in those without such insertions (Al et al., 2022). Additionally, studies have reported that over 70% of individuals with respiratory-onset ALS harbor *ERBB4* insertions, compared to 25% in the general population, indicating that structural variations in *ERBB4* may also influence the site of disease onset (Al et al., 2022). While patients carrying *ERBB4* variants rarely exhibit cognitive dysfunction, these variants have been identified in individuals with FTD and ALS-FTD (Dols-Icardo et al., 2018; Gromicho et al., 2020; Sun et al., 2020; Tábuas-Pereira et al., 2022; Cai et al., 2024), suggesting that the role of *ERBB4* in cognitive impairment and neurodegenerative diseases warrants further investigation.

Rare *ERBB4* variants are infrequently observed among ALS patients, and most missense variants in *ERBB4* are likely non-pathogenic, yet the NRG1-ERBB4 axis holds significant potential for monitoring ALS progression and serving as a therapeutic target. The presence of circulating ERBB4 ectodomain fragments (specifically 55 kDa and 80 kDa) in the cerebrospinal fluid and plasma of both ALS and ALS-FTD patients has been confirmed, with levels found to be decreased compared to controls and absent in the plasma of *ERBB4* knockout mice (Lopez-Font et al., 2019). This indicates a potential tool to evaluate the impairment of the ERBB4 pathway and suggests it may serve as a useful biomarker in ALS. Interestingly, disruption of the NRG1/ERBB4 axis holds therapeutic potential for both cancer and ALS. In cancer, suppression of this axis is being tested with promising results in patients with tumors harboring NRG1 fusions or similar alterations causing aberrant activation or expression (Adashek et al., 2024). In ALS, while the interactions appear more complex, evidence suggests that reduced ERBB4 function may upregulate NRG1, leading to secondary overstimulation of other ERBB receptors (Song et al., 2012). This highlights the potential for repurposing pan-ERBB/HER inhibitors, already approved and in use for cancer, as a therapeutic strategy in ALS.

This study has several limitations that should be acknowledged. First, the lack of functional validation for the identified *ERBB4* variants limits the ability to confirm their pathogenicity and clarify their mechanistic role in ALS pathogenesis. Second, the absence of longitudinal follow-up data in the included studies hinders the evaluation of long-term clinical progression in ALS patients with *ERBB4* variants and the establishment of robust genotype–phenotype correlations. Third, inconsistencies in the application of ACMG classification criteria across studies may introduce bias, complicating cross-study comparisons and pooled mutation frequency analyses.

This comprehensive analysis of ERBB4 variants in ALS provides important insights into their frequency, distribution, and clinical implications. While ERBB4 mutations are relatively rare, occurring in less than 1% of ALS patients globally, their impact on disease onset and ethnic-specific patterns warrant attention in genetic screening protocols. The identification of novel variants and their potential functional consequences, coupled with the emerging understanding of the NRG1-ERBB4 signaling pathway’s role in motor neuron survival, suggests promising therapeutic opportunities. Future studies incorporating functional validation and longitudinal clinical data will be crucial to fully elucidate the role of ERBB4 in ALS pathogenesis and its potential as a therapeutic target.

## Data Availability

The datasets presented in this study can be found in online repositories. The names of the repository/repositories and accession number(s) can be found in the article/Supplementary material.

## Footnotes

^1^ http://www.mbiobank.com/

^2^ http://umd.be/Redirect.html

^3^ https://spliceailookup.broadinstitute.org

^4^ https://rddc.tsinghua-gd.org/tool/pathogenicity

^5^ https://varseak.bio/

^6^ http://www.cebm.brown.edu/openmeta/index.html
