# Supplementary material for: Genetic analysis of ERBB4 gene in Chinese patients with amyotrophic lateral sclerosis: a single-center study and systematic review of published literature
Source: Front Aging Neurosci. 2025 May 21;17:1584541. doi: 10.3389/fnagi.2025.1584541 (PMC12133825; doi:10.3389/fnagi.2025.1584541)
Supplement: Supplementary file 1 [file Data_Sheet_1.pdf]

Table 1. Summary of rare variants of *ERBB4* associated with ALS

ERBB4 Variants	Age	Gender	Onset	sALS/fALS/ALS-FTD	Multilple variants	Prediction	country_author_pulication year[ALS with ERBB4 variants/total patients numbers]
c.11C>A p.A4E	NA	NA	NA	NA	No	VUS	USA_Goutman, S. A. 2024 [75/7490]
c.19C>T p.L7F	NA	NA	NA	NA	No	VUS	China_Chen, W. 2020 [11/268]
c.35G>A p.S12N	NA	NA	NA	sALS	No	VUS	Germany_Ruf, W. p. 2023 [19/2267]
c.36C>A p.S12R	NA	NA	NA	NA	No	VUS	USA_Goutman, S. A. 2024 [75/7535]
c.82+8C>T	42	M	Limb	sALS	No	Likely benign	China_Shen, D. 2024 [23/1672]
c.139C>T p.R47*	NA	NA	NA	NA	No	VUS	USA_Goutman, S. A. 2024 [75/7501]
c.157T>C p.Y53H	NA	NA	NA	sALS	No	VUS	Multi-centers_Van Daele, S. H. 2023[31/6103]
c.158A>G p.Y53C	NA	NA	NA	sALS	No	VUS	Multi-centers_Van Daele, S. H. 2023[31/6103]/Turkey_Tunca, C. 2020 [5/1200]
c.206A>T p.E69V	50	M	Limb	sALS	No	Benign	India_Narain, p. 2018/2019 [2/154]
c.213C>G p.N71K	NA	NA	NA	NA	No	VUS	USA_Goutman, S. A. 2024 [75/7505]
c.268G>T p.A90S	NA	NA	NA	sALS	No	VUS	Germany_Ruf, W. p. 2023 [19/2267]
c.268G>T p.A90S	NA	NA	NA	NA	No	VUS	Hungary_Tripolszki, K. 2019 [1/107]
c.268G>T p.A90S	NA	NA	NA	sALS	No	VUS	Multi-centers_Van Daele, S. H. 2023[31/6103]
c.269C>T p.A90V	NA	NA	NA	NA	No	VUS	USA_Goutman, S. A. 2024 [75/7551]
c.284G>A p.R95H	41	F	Limb	sALS	No	VUS	China_Wang, F. 2022 [16/448]
c.284G>A p.R95H	NA	NA	NA	NA	No	Benign	China_Chen, W. 2020 [11/268]
c.308G>A p.R103H	68	F	Bulbar	sALS	No	Pathogenic	India_Narain, p. 2018/2019 [2/154]
c.308G>A p.R103H	NA	NA	NA	NA	***SOD1* c.435G>C p.L145F**	Likely Pathogenic	Italy_Grassano, M. 2022 [9/1043]
c.308G>A p.R103H	NA	NA	NA	sALS	No	VUS	Multi-centers_Van Daele, S. H. 2023[31/6103]/Turkey_Tunca, C. 2020 [5/1200]
c.308G>A p.R103H	NA	NA	NA	NA	No	VUS	USA_Goutman, S. A. 2024 [75/7491]
c.317G>A p.R106H	57	M	Limb	sALS	No	Likely pathogenic	China_Wang, F. 2022 [16/448]
c.328C>G p.L110V	NA	NA	NA	NA	No	VUS	USA_Goutman, S. A. 2024 [75/7553]
c.392T>C p.L131P	NA	NA	NA	NA	No	VUS	USA_Goutman, S. A. 2024 [75/7557]
c.472G>A p.A158T	NA	NA	NA	NA	No	VUS	USA_Goutman, S. A. 2024 [75/7488]
c.421+5G>A	NA	NA	NA	NA	No	VUS	Italy_Pensato, V. 2020 [2/213]
c.491A>C p.Q164	43	M	Limb	sALS	No	Likely pathogenic	China_Wang, F. 2022 [16/448]
c.502C>T p.R168W	NA	NA	NA	NA	No	VUS	USA_Goutman, S. A. 2024 [75/7496]
c.503G>A p.R168Q	NA	NA	NA	NA	No	VUS	USA_Goutman, S. A. 2024 [75/7495]
c.532G>C p.V178L	NA	NA	NA	NA	No	Likely Pathogenic	Italy_Grassano, M. 2022 [9/1043]
c.553G>A p.G185R	NA	NA	NA	NA	No	VUS	USA_Goutman, S. A. 2024 [75/7511]
c.562C>T p.R188C	NA	NA	NA	sALS	No	VUS	Germany_Ruf, W. p. 2023 [19/2267]
c.563G>A p.R188H	NA	NA	NA	NA	No	VUS	USA_Goutman, S. A. 2024 [75/7497]
c.580A>G p.T194A	NA	NA	NA	NA	No	VUS	USA_Goutman, S. A. 2024 [75/7537]
c.586C>T p.R196C	NA	NA	NA	NA	No	Likely Pathogenic	Italy_Grassano, M. 2022 [9/1043]
c.587G>A p.R196H	NA	NA	NA	NA	No	VUS	USA_Goutman, S. A. 2024 [75/7498]
c.610C>G p.H204D	47	M	Limb	sALS	No	VUS	China_Shen, D. 2024 [23/1672]
c.634G>T p.V212	48	M	Bulbar	fALS	No	Likely pathogenic	China_Wang, F. 2022 [16/448]
c.655G>A p.G219S	51	M	Limb	sALS	No	VUS	China_Shen, D. 2024 [23/1672]
c.655G>A p.G219S	NA	NA	NA	sALS	No	VUS	Multi-centers_Van Daele, S. H. 2023[31/6103]/Turkey_Tunca, C. 2020 [5/1200]
c.710G>A p.G237D	NA	NA	NA	NA	No	VUS	USA_Goutman, S. A. 2024 [75/7512]
c.794C>A p.T265N	NA	NA	NA	NA	No	VUS	USA_Goutman, S. A. 2024 [75/7559]
c.812C>T p.T271I	NA	NA	NA	fALS	*KIF5A* c.2957C>T p.P986L	VUS	Germany_Müller, K. 2018 [3/301]/Brenner, D. 2018
c.826G>C p.E276Q	NA	NA	NA	sALS	No	VUS	Multi-centers_Van Daele, S. H. 2023[31/6103]
c.829C>T p.H277Y	NA	NA	NA	NA	No	VUS	USA_Goutman, S. A. 2024 [75/7544]
c.842C>G p.A281G	NA	NA	NA	NA	No	VUS	USA_Goutman, S. A. 2024 [75/7489]
c.847T>C p.Y283H	NA	NA	NA	sALS	No	VUS	Germany_Ruf, W. p. 2023 [19/2267]
c.847T>C p.Y283H	NA	NA	NA	NA	No	VUS	USA_Goutman, S. A. 2024 [75/7541]
c.850A>G p.T284A	NA	NA	NA	sALS	No	VUS	Multi-centers_Van Daele, S. H. 2023[31/6103]
c.883C>T p.H295Y	NA	NA	NA	NA	No	VUS	USA_Goutman, S. A. 2024 [75/7515]
c.884-7_884-6insTT	49	M	Bulbar	sALS	No	VUS	China_Shen, D. 2024 [23/1672]
c.884-7T>A	57	M	Limb	sALS	No	VUS	China_Shen, D. 2024 [23/1672]
c.891T>G p.F297L	NA	NA	NA	NA	No	VUS	USA_Goutman, S. A. 2024 [75/7532]
c.917G>A p.R306H	NA	NA	NA	NA	No	VUS	USA_Goutman, S. A. 2024 [75/7499]
c.919G>T p.A307S	44	M	Limb	sALS	No	VUS	Turkey_Gorukmez, O. 2023[1589 casese with suspected rare genetic disorders]
c.965T>A p.M322K	NA	NA	NA	NA	No	VUS	China_Liu, Z. 2019 [1/45]
c.965T>A p.M322K	NA	NA	NA	NA	No	VUS	China_Chen, Y. 2022 [9/1587]
c.974C>T p.P325L	68	F	Limb	sALS	No	VUS	China_Shen, D. 2024 [23/1672]
c.974C>T p.P325L	NA	NA	NA	NA	No	Damaging	China_Chen, Y. 2022 [9/1587]
c.1009A>G p.I337V	NA	NA	NA	NA	No	VUS	USA_Goutman, S. A. 2024 [75/7519]
c.1069A>G p.I357V	NA	NA	NA	NA	No	VUS	USA_Goutman, S. A. 2024 [75/7520]
c.1122T>G p.H374Q	NA	NA	NA	ALS-FTD	No	VUS	Spain_Dols-Icardo, O. 2018 [2/54]
c.1122T>G p.H374Q	NA	NA	NA	sALS	No	Tolerated	Malta_Borg, R. 2021/Farrugia Wismayer, M. 2023 [3/52]
c.1171G>A p.V391I	NA	NA	NA	NA	No	VUS	USA_Goutman, S. A. 2024 [75/7545]
c.1177C>T p.R393W	NA	NA	NA	sALS	No	VUS	Multi-centers_Van Daele, S. H. 2023[31/6103]
c.1178G>A p.R393Q	NA	NA	NA	NA	No	VUS	USA_Goutman, S. A. 2024 [75/7500]
c.1240T>C p.F414L	71	M	Limb	ALS-FTD	No	VUS	Portugal_Gromicho, M. 2020 [2/34]
c.1328C>T p.T443I	NA	NA	NA	NA	No	VUS	USA_Goutman, S. A. 2024 [75/7538]
c.1378A>C p.I460L	NA	NA	NA	NA	No	VUS	USA_Goutman, S. A. 2024 [75/7521]
c.1395C>A p.N465K	NA	NA	NA	NA	No	VUS	UK_SheFard, S. R. 2021 [1/100]
c.1395C>A p.N465K	NA	NA	NA	NA	No	VUS	USA_Goutman, S. A. 2024 [75/7504]
c.1431_1432del p.F478Q*fs*	NA	NA	NA	NA	No	VUS	USA_Goutman, S. A. 2024 [75/7546]
c.1441A>G p.I481V	70	NA	Bulbar	fALS	No	VUS	Italy_Pensato, V. 2020 [2/213]
c.1490-3C>T	51	M	Bulbar	sALS	*HNRNPA1* c.847G>A p.G283R	Likely benign	China_Shen, D. 2024 [23/1672]
c.1490-3C>T	27	F	Limb	sALS	No	Likely benign	China_Shen, D. 2024 [23/1672]
c.1490-3C>T	38	M	Limb	sALS	*OPTN* c.1634G>A p.R545Q	Likely benign	China_Shen, D. 2024 [23/1672]
c.1490-5C>G	49	M	Limb	sALS	No	VUS	China_Shen, D. 2024 [23/1672]
c.1514A>T p.H505L	NA	NA	NA	NA	No	VUS	USA_Goutman, S. A. 2024 [75/7516]
c.1573C>T p.R525C	NA	NA	NA	sALS	No	VUS	Multi-centers_Van Daele, S. H. 2023[31/6103]
c.1573C>T p.R525C	NA	NA	NA	NA	No	VUS	USA_Goutman, S. A. 2024 [75/7547]
c.1616A>C p.Y539S	NA	NA	NA	NA	No	Damaging	China_Chen, Y. 2022 [9/1587]
c.1624G>A p.E542K	NA	NA	NA	NA	No	VUS	China_Chen, W. 2020 [11/268]
c.1624G>A p.E542K	NA	NA	NA	NA	No	VUS	China_Chen, W. 2020 [11/268]
c.1630C>T p.R544W	NA	F	NA	No symptom	No	Likely pathogenic	Spain_Capalbo, A. 2019 [14125 individuals undergoing preconception carrier screening]
c.1658T>G p.V553G	NA	NA	NA	NA	No	Damaging	China_Chen, Y. 2022 [9/1587]
c.1662G>C p.E554D	NA	NA	NA	NA	No	VUS	USA_Goutman, S. A. 2024 [75/7508]
c.1708C>T p.H570Y	NA	NA	NA	sALS	No	VUS	Germany_Ruf, W. p. 2023 [19/2267]
c.1709A>G p.H570R	NA	NA	NA	NA	No	VUS	USA_Goutman, S. A. 2024 [75/7517]
c.1712G>C p.G571A	NA	NA	NA	NA	No	VUS	USA_Goutman, S. A. 2024 [75/7548]
c.1717G>A p.G573S	NA	NA	NA	NA	No	VUS	USA_Goutman, S. A. 2024 [75/7513]
c.1718G>A p.G573D	NA	NA	NA	sALS	No	Pathogenic	Germany_Ruf, W. p. 2023 [19/2267]
c.1720C>A p.P574T	NA	NA	NA	sALS	No	VUS	Germany_Ruf, W. p. 2023 [19/2267]
c.1720C>A p.P574T	NA	NA	NA	sALS	No	VUS	Multi-centers_Van Daele, S. H. 2023[31/6103]
c.1720C>A p.P574T	NA	NA	NA	NA	No	VUS	USA_Goutman, S. A. 2024 [75/7533]
c.1728C>G p.N576K	NA	NA	NA	NA	No	VUS	USA_Goutman, S. A. 2024 [75/7549]
c.1772A>G p.E591G	NA	NA	NA	NA	*ANXA11* c.C922T p.R308X	Likely Pathogenic	Italy_Grassano, M. 2022 [9/1043]
c.1829C>G p.P610R	NA	NA	NA	NA	No	VUS	USA_Goutman, S. A. 2024 [75/7534]
c.1834C>T p.R612W	NA	NA	NA	sALS	No	VUS	Multi-centers_Van Daele, S. H. 2023[31/6103]
c.1835G>A p.R612Q	NA	NA	NA	NA	No	VUS	USA_Goutman, S. A. 2024 [75/7502]
c.1879G>A p.G627S	NA	NA	NA	sALS	***SOD1* c.262G>A p.V88M**	VUS	Germany_Ruf, W. p. 2023 [19/2267]
c.1891C>T p.H631Y	NA	NA	NA	NA	No	VUS	USA_Goutman, S. A. 2024 [75/7518]
c.1898G>C p.C633S	NA	NA	NA	NA	No	Likely Pathogenic	Italy_Grassano, M. 2022 [9/1043]
c.1912T>C p.W638R	NA	NA	NA	NA	No	Likely Pathogenic	Italy_Grassano, M. 2022 [9/1043]
c.1913G>A p.W638*	NA	NA	NA	NA	No	Likely Pathogenic	Italy_Grassano, M. 2022 [9/1043]
c.1927A>G p.T643A	NA	NA	NA	NA	No	Benign	China_Chen, W. 2020 [11/268]
c.1928C>T p.T643I	NA	NA	NA	sALS	*SPAST* c.125C>A p.P42H	Tolerated	USA_Gibson, S. B. 2017 [3/87]
c.1967G>A p.G656E	NA	NA	NA	NA	No	VUS	China_Chen, W. 2020 [11/268]
c.1972A>T p.I658F	38	F	Bulbar	sALS	No	VUS	China_Zhang, N. 2023 [case report]
c.1972A>T p.I658F	57	F	Bulbar	sALS	No	Benign	China_Wang, F. 2022 [16/448]
c.1972A>T p.I658F	50	F	Limb	fALS	No	Benign	China_Wang, F. 2022 [16/448]
c.1972A>T p.I658F	35	M	Limb	sALS	No	Benign	China_Wang, F. 2022 [16/448]
c.1972A>T p.I658F	44	F	Limb	sALS	No	Benign	China_Wang, F. 2022 [16/448]
c.1972A>T p.I658F	55	F	Limb	sALS	No	Benign	China_Wang, F. 2022 [16/448]
c.1972A>T p.I658F	43	F	Limb	sALS	No	Likely benign	China_Shen, D. 2024 [23/1672]
c.1972A>T p.I658F	44	M	Bulbar	sALS	*SPG11* c.581C>T p.P194L	Likely benign	China_Shen, D. 2024 [23/1672]
c.1981C>G p.L661V	NA	NA	NA	NA	No	VUS	USA_Goutman, S. A. 2024 [75/7550]
c.1997T>C p.I666T	36	M	Bulbar	ALS-FTD	No	Possible pathogenic	Spain_Dols-Icardo, O. 2018 [2/54]
c.1997T>C p.I666T	NA	NA	NA	NA	No	VUS	USA_Goutman, S. A. 2024 [75/7522]
c.2000T>C p.V667A	36	M	Limb	fALS	No	Benign	China_Wang, F. 2022 [16/448]
c.2079+5G>A	NA	NA	NA	NA	No	VUS	USA_Goutman, S. A. 2024 [75/7483]
c.2079+6G>A	NA	NA	NA	NA	No	VUS	USA_Goutman, S. A. 2024 [75/7486]
c.2080-3C>T	NA	NA	NA	NA	No	VUS	USA_Goutman, S. A. 2024 [75/7484]
c.2087A>C p.E696A	NA	NA	NA	sALS	No	VUS	Germany_Ruf, W. p. 2023 [19/2267]
c.2087A>C p.E696A	NA	NA	NA	NA	No	VUS	USA_Goutman, S. A. 2024 [75/7509]
c.2116A>G p.N706D	53	M	Limb	sALS	No	Pathogenic	Korea_Kwon, Y. 2024 [case report]
c.2117A>G p.N706S	NA	NA	NA	sALS	No	VUS	Multi-centers_Van Daele, S. H. 2023[31/6103]
c.2132G>A p.R711H	NA	NA	NA	sALS	No	VUS	Multi-centers_Van Daele, S. H. 2023[31/6103]
c.2136T>G p.I712M	55	F	Limb	ALS-FTD	No	Possible pathogenic	China_Sun, L. 2020 [case report-1/84 dementia patients]
c.2192C>T p.T731M	NA	NA	NA	NA	No	VUS	USA_Goutman, S. A. 2024 [75/7539]
c.2204G> T p.G735V	NA	NA	NA	sALS	No	Damaging	USA_Gibson, S. B. 2017 [3/87]
c.2210G>C p.W737S	NA	NA	NA	NA	No	VUS	USA_Goutman, S. A. 2024 [75/7540]
c.2281G>A p.A761T	NA	NA	NA	NA	No	Damage	China_Liu, Z. 2021 [2/753]
c.2296A>G p.M766V	NA	NA	NA	sALS	No	VUS	Multi-centers_Van Daele, S. H. 2023[31/6103]
c.2324T>C p.M775T	NA	NA	NA	sALS	No	VUS	Multi-centers_Van Daele, S. H. 2023[31/6103]
c.2342T>C p.V781A	NA	NA	NA	NA	No	Pathogenic	China_Chen, W. 2020 [11/268]
c.2345G>C p.R782P	43	F	Limb	sALS	No	Likely Pathogenic	China_Shen, D. 2024 [23/1672]
c.2395A>G p.M799V	NA	NA	NA	NA	No	Damage	China_Liu, Z. 2021 [2/753]

c.2395A>G p.M799V	NA	NA	NA	NA	No	Damaging	China_Chen, Y. 2022 [9/1587]
c.2396T>C p.M799T	38	F	Limb	sALS	No	Likely Pathogenic	China_Shen, D. 2024 [23/1672]
c.2397G>A p.M799I	NA	NA	NA	NA	No	VUS	USA_Goutman, S. A. 2024 [75/7528]
c.2428G>A p.E810K	NA	NA	NA	fALS	No	VUS	Germany_Müller, K. 2018 [3/301]
c.2444T>C p.I815T	NA	NA	NA	NA	No	VUS	USA_Goutman, S. A. 2024 [75/7523]
c.2446G>A p.G816R	NA	NA	NA	NA	No	Damaging	China_Chen, Y. 2022 [9/1587]
c.2495T>G p.M832R	NA	NA	NA	sALS	No	VUS	Germany_Ruf, W. p. 2023 [19/2267]
c.2513G>A p.R838Q	NA	NA	NA	sALS	No	VUS	Multi-centers_Van Daele, S. H. 2023[31/6103]
c.2518G>A p.V840I	NA	NA	NA	sALS	No	VUS	Multi-centers_Van Daele, S. H. 2023[31/6103]/Turkey_Tunca, C. 2020 [5/1200]
c.2524C>T p.R842W	NA	NA	NA	sALS	No	VUS	Multi-centers_Van Daele, S. H. 2023[31/6103]
c.2525G>A p.R842Q	NA	NA	NA	sALS	No	VUS	Multi-centers_Van Daele, S. H. 2023[31/6103]
c.2525G>A p.R842Q	NA	NA	NA	NA	No	Likely Pathogenic	Italy_Grassano, M. 2022 [9/1043]
c.2539C>T p.R847C	49	M	Limb	sALS	No	Likely Pathogenic	China_Shen, D. 2024 [23/1672]
c.2554A>G p.K852E	NA	NA	NA	sALS	No	VUS	Multi-centers_Van Daele, S. H. 2023[31/6103]
c.2639G>A p.G880E	NA	NA	NA	sALS	No	VUS	Germany_Ruf, W. p. 2023 [19/2267]
c.2661G>A p.M887I	NA	NA	NA	NA	No	VUS	USA_Goutman, S. A. 2024 [75/7529]
c.2674A>G p.I892V	NA	NA	NA	NA	No	VUS	UK_Leighton, D. J. 2024 [1/339]
c.2674A>G p.I892V	NA	NA	NA	NA	No	VUS	USA_Goutman, S. A. 2024 [75/7524]
c.2707G>A p.V903I	39	M	Limb	fALS	No	VUS	China_Wang, F. 2022 [16/448]
c.2728A>G p.I910V	59	M	Limb	ALS-FTD	No	VUS	Portugal_Gromicho, M. 2020 [2/34]
c.2742G>T p.M914I	NA	NA	NA	NA	No	VUS	USA_Goutman, S. A. 2024 [75/7530]
c.2780G>A p.R927Q	63	M	Limb	fALS	No	Pathogenic	Japan_Takahashi, Y. 2013 [case report+2/1182]
c.2780G>A p.R927Q	67	M	Limb	fALS	No	Pathogenic	Japan_Takahashi, Y. 2013 [case report+2/1182]
c.2813G>A p.R938H	NA	NA	NA	sALS	No	VUS	Multi-centers_Van Daele, S. H. 2023[31/6123]
c.2830A>G p. I944V	55	F	Bulbar	sALS	No	VUS	China_Shen, D. 2024 [23/1672]
c.2845G>A p.V949I	NA	NA	NA	NA	No	VUS	USA_Goutman, S. A. 2024 [75/7543]
c.2867-5T>A	NA	NA	NA	NA	No	VUS	USA_Goutman, S. A. 2024 [75/7485]
c.2935C>G p.R979G	53	M	Limb	sALS	No	VUS	China_Shen, D. 2024 [23/1672]
c.2948G>T p.R983I	NA	NA	NA	sALS	No	VUS	Multi-centers_Van Daele, S. H. 2023[31/6103]
c.2956G>C p.V986L	NA	NA	NA	sALS	No	VUS	Multi-centers_Van Daele, S. H. 2023[31/6103]
c.2975G>A p.R992H	46	M	Limb	sALS	No	VUS	China_Wang, F. 2022 [16/448]
c.2975G>A p.R992H	NA	NA	NA	NA	No	VUS	USA_Goutman, S. A. 2024 [75/7552]
c.2989A>C p.S997R	51	M	Limb	sALS	No	Likely Pathogenic	China_Shen, D. 2024 [23/1672]
c.3045T>G p.D1015E	46	F	Bulbar	sALS	No	VUS	China_Shen, D. 2024 [23/1672]
c.3050T>C p.M1017T	NA	NA	NA	NA	No	VUS	USA_Goutman, S. A. 2024 [75/7526]
c.3107C>G p.T1036S	NA	NA	NA	NA	No	VUS	China_Chen, W. 2020 [11/268]
c.3136-5A>G	18	F	Limb	sALS	No	VUS	China_Shen, D. 2024 [23/1672]
c.3136-5A>G	NA	NA	NA	NA	No	VUS	China_Chen, W. 2020 [11/268]
c.3143T>C p.I1048T	40	M	NA	fALS	No	VUS	China_Wang, F. 2022 [16/448]
c.3153C>A p.S1051R	NA	NA	NA	sALS	No	VUS	Germany_Ruf, W. p. 2023 [19/2267]
c.3176T>C p.M1059T	NA	NA	NA	sALS	No	Tolerated	Malta_Borg, R. 2021/Farrugia Wismayer, M. 2023 [3/52]
c.3176T>C p.M1059T	NA	NA	NA	NA	No	VUS	USA_Goutman, S. A. 2024 [75/7527]
c.3182G>A p.G1061E	NA	NA	NA	sALS	No	VUS	Germany_Ruf, W. p. 2023 [19/2267]
c.3182G>A p.G1061E	NA	NA	NA	NA	No	VUS	USA_Goutman, S. A. 2024 [75/7510]
c.3186C>A p.N1062K	NA	NA	NA	NA	No	VUS	USA_Goutman, S. A. 2024 [75/7503]
c.3189G>C p.Q1063H	NA	NA	NA	sALS	No	VUS	Germany_Ruf, W. p. 2023 [19/2267]
c.3206G>T p.G1069V	NA	NA	NA	sALS	No	VUS	Multi-centers_Van Daele, S. H. 2023[31/6103]
c.3238C>A p.P1080T	NA	NA	NA	sALS	No	VUS	Germany_Ruf, W. p. 2023 [19/2267]
c.3253A>G p.T1085A	NA	NA	NA	sALS	No	Tolerated	USA_Gibson, S. B. 2017 [3/87]
c.3253A>G p.T1085A	NA	NA	NA	NA	No	VUS	USA_Goutman, S. A. 2024 [75/7536]
c.3287G>A p.G1096D	NA	NA	NA	sALS	No	VUS	Multi-centers_Van Daele, S. H. 2023[31/6103]
c.3287delG p.G1096*fs*56	NA	NA	NA	sALS	No	Pathogenic	Germany_Ruf, W. p. 2023 [19/2267]
c.3334C>T p.R1112C	48	NA	NA	fALS	No	Likely pathogenic	Turkey_Tunca, C. 2020 [5/1200]
c.3335G>A p.R1112H	NA	NA	NA	NA	No	VUS	USA_Goutman, S. A. 2024 [75/7492]
c.3337A>G p.K1113E	50+	NA	Limb	sALS	No	VUS	Norway_Olsen, C. G. 2022 [2/279]
c.3337A>G p.K1113E	NA	NA	NA	fALS	***C9orf72***	VUS	Norway_Olsen, C. G. 2022 [2/279]
c.3341C>A p.P1114Q	NA	NA	NA	sALS	No	VUS	Germany_Ruf, W. p. 2023 [19/2267]
c.3355G>A p.V1119I	37	F	Limb	sALS	No	VUS	China_Wang, F. 2022 [16/448]
c.3371G>A p.S1124N	59	F	Limb	sALS	***SOD1* c.37G>C p.G13R**	VUS	China_Shen, D. 2024 [23/1672]
c.3400G>A p.V1134M	NA	NA	NA	NA	No	VUS	China_Chen, W. 2020 [11/268]
c.3400G>A p.V1134M	NA	NA	NA	NA	No	VUS	USA_Goutman, S. A. 2024 [75/7542]
c.3400G>T p.V1134L	NA	NA	NA	NA	No	VUS	Italy_Lamp, M.2018 [1/76]
c.3427G>A p.G1143R	NA	NA	NA	sALS	No	VUS	Multi-centers_Van Daele, S. H. 2023[31/6103]
c.3433C>A p.L1145M	NA	NA	NA	NA	No	VUS	USA_Goutman, S. A. 2024 [75/7554]
c.3446G>T p.G1149V	50	NA	Limb	sALS	*TARDBP* c.859G>A p.G287S<br>*NEK1* c.304G>T p.E102*<br>*VEGFA* c.1015C>T p.R339W	VUS	Germany_Yilmaz, R. 2022 [case report]
c.3446G>T p.G1149V	NA	NA	NA	NA	No	Likely Pathogenic	Italy_Grassano, M. 2022 [9/1043]
c.3446G>T p.G1149V	NA	NA	NA	sALS	No	VUS	Germany_Ruf, W. p. 2023 [19/2267]
c.3446G>T p.G1149V	NA	NA	NA	sALS	No	VUS	Multi-centers_Van Daele, S. H. 2023[31/6103]
c.3464G>A p.R1155Q	52	M	Limb	fALS	No	VUS	China_Shen, D. 2024 [23/1672]
c.3464G>A p.R1155Q	NA	NA	NA	NA	No	VUS	China_Chen, Y. 2022 [9/1587]
c.3464G>A p.R1155Q	NA	NA	NA	NA	No	VUS	China_Chen, Y. 2022 [9/1587]
c.3494C>G p.P1165R	NA	NA	NA	sALS	No	VUS	Germany_Ruf, W. p. 2023 [19/2267]
c.3521G>A p.R1174Q	49	M	Limb	fALS	No	VUS	China_Shen, D. 2024 [23/1672]
c.3527A>T p.K1176I	NA	NA	NA	NA	No	VUS	USA_Goutman, S. A. 2024 [75/7525]
c.3565C>T p.H1189Y	NA	NA	NA	NA	No	VUS	USA_Goutman, S. A. 2024 [75/7514]
c.3604T>G p.Y1202D	NA	NA	NA	sALS	No	VUS	Multi-centers_Van Daele, S. H. 2023[31/6103]
c.3613G>A p.E1205K	NA	NA	NA	NA	No	VUS	USA_Goutman, S. A. 2024 [75/7506]
c.3635T>C p.F1212S	NA	NA	NA	NA	No	VUS	USA_Goutman, S. A. 2024 [75/7531]
c.3654A>T p.K1218N	62	M	Limb	sALS	No	Benign	China_Wang, F. 2022 [16/448]
c.3656C>G p.A1219G	NA	NA	NA	NA	No	VUS	USA_Goutman, S. A. 2024 [75/7555]
c.3685A>G p.M1229V	NA	NA	NA	NA	No	VUS	China_Chen, W. 2020 [11/268]
c.3707C>T p.A1236V	NA	NA	NA	NA	No	VUS	USA_Goutman, S. A. 2024 [75/7487]
c.3769G>C p.D1257H	36	M	Limb	sALS	No	VUS	China_Wang, F. 2022 [16/448]
c.3809A>G p.Q1270R	NA	NA	NA	fALS	No	VUS	Germany_Müller, K. 2018 [3/301]
c.3814G>A p.G1272R	NA	NA	NA	sALS	No	Damaging	Malta_Borg, R. 2021/Farrugia Wismayer, M. 2023 [3/52]
c.3817C>T p.R1273W	NA	NA	NA	NA	No	VUS	China_Chen, Y. 2022 [9/1587]
c.3823C>T p.R1275W	45	F	Limb	sALS	No	Pathogenic	Japan_Takahashi, Y. 2013 [case report+2/1182]
c.3823C>T p.R1275W	NA	NA	NA	sALS	No	VUS	Multi-centers_Van Daele, S. H. 2023[31/6103]
c.3823C>T p.R1275W	NA	NA	NA	NA	No	Pathogenic	USA_Goutman, S. A. 2024 [75/7494]
c.3824G>A p.R1275Q	NA	NA	NA	sALS	No	VUS	Multi-centers_Van Daele, S. H. 2023[31/6103]
c.3824G>A p.R1275Q	NA	NA	NA	NA	No	VUS	USA_Goutman, S. A. 2024 [75/7493]
c.3861G>T p.E1287D	NA	NA	NA	sALS	No	VUS	Multi-centers_Van Daele, S. H. 2023[31/6103]
c.3861G>T p.E1287D	NA	NA	NA	NA	No	VUS	USA_Goutman, S. A. 2024 [75/7507]

Table 2. Summary of cases carrying rare variants in both *ERBB4* and other ALS-related genes

ERBB4 Variants	Age	Gender	Onset	sALS/fALS	Multilple variants	Prediction	country_author_pulication year[ALS with ERBB4 variants/total patients numbers]
c.1772A>G p.E591G	NA	NA	NA	NA	*ANXA11* c.C922T p.R308X	Likely Pathogenic	Italy_Grassano, M. 2022 [9/1043]
c.3337A>G p.K1113E	NA	NA	NA	fALS	***C9orf72***	VUS	Norway_Olsen, C. G. 2022 [2/279]
c.1490-3C>T	51	M	Bulbar	sALS	*HNRNPA1* c.847G>A p.G283R	Likely benign	China_Shen, D. 2024 [23/1672]
c.812C>T p.T271I	NA	NA	NA	fALS	*KIF5A* c.2957C>T p.P986L	VUS	Germany_Müller, K. 2018 [3/301]/Brenner, D. 2018
c.1490-3C>T	38	M	Limb	sALS	*OPTN* c.1634G>A p.R545Q	Likely benign	China_Shen, D. 2024 [23/1672]
c.1879G>A p.G627S	NA	NA	NA	sALS	***SOD1* c.262G>A p.V88M**	VUS	Germany_Ruf, W. p. 2023 [19/2267]
c.3371G>A p.S1124N	59	F	Limb	sALS	***SOD1* c.37G>C p.G13R**	VUS	China_Shen, D. 2024 [23/1672]
c.308G>A p.R103H	NA	NA	NA	NA	***SOD1* c.435G>C p.L145F**	Likely Pathogenic	Italy_Grassano, M. 2022 [9/1043]
c.1928C>T p.T643I	NA	NA	NA	sALS	*SPAST* c.125C>A p.P42H	Tolerated	USA_Gibson, S. B. 2017 [3/87]
c.1972A>T p.I658F	44	M	Bulbar	sALS	*SPG11* c.581C>T p.P194L<br>*TARDBP* c.859G>A p.G287S<br>*NEK1* c.304G>T p.E102*	Likely benign	China_Shen, D. 2024 [23/1672]
c.3446G>T p.G1149V	50	NA	Limb	sALS	*VEGFA* c.1015C>T p.R339W	VUS	Germany_Yilmaz, R. 2022 [case report]

Figure 1 The chromatograph of *ERBB4* c.1490-3C>T and OPTN c.1634G>A in an ALS patient and family verification

Sample	Result	*ERBB4*	chr2:212543912	c.1490-3C>T	-
Patient	Het	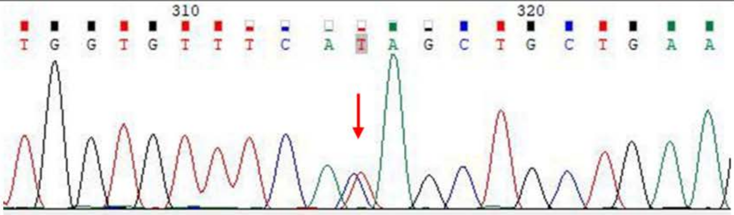			
Father	N	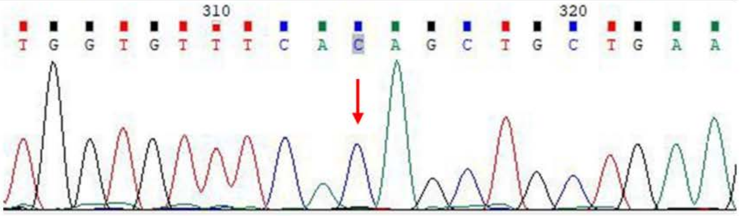			
Mother	Het	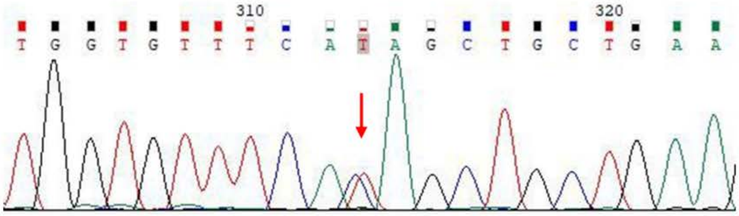			

Sample	Result	*OPTN*	chr10:13178766	c.1634G>A	p.Arg545Gln
Patient	Het	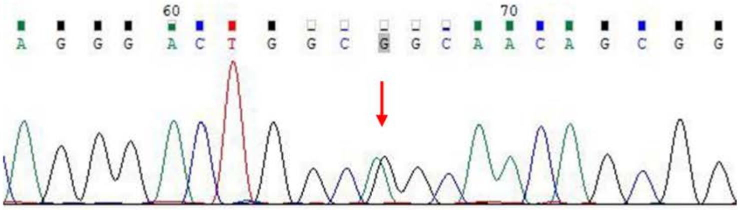			
Father	N	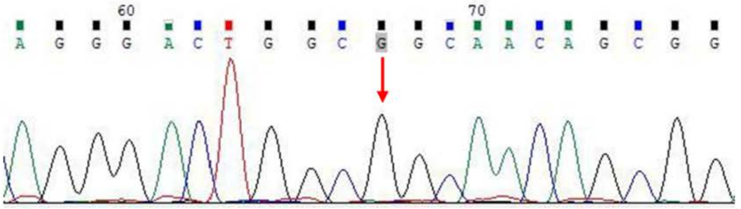			
Mother	Het	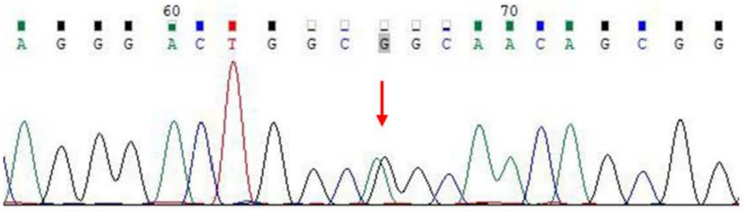			

Figure 2 mRNA expression of *ERBB4* c. 1490-3C>T

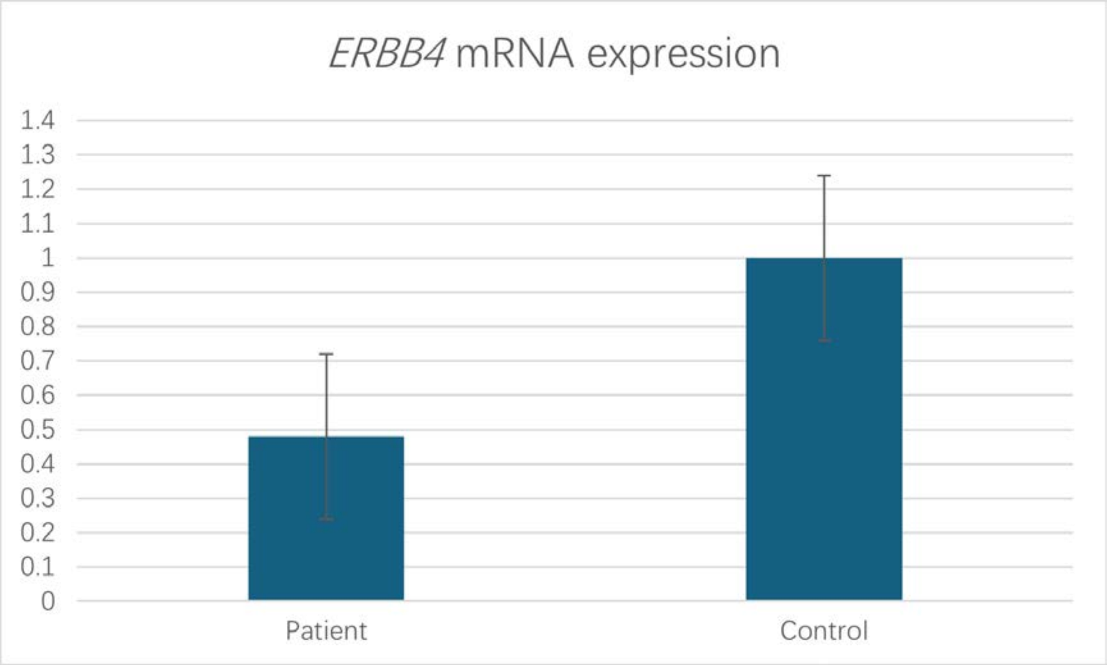

The relative mRNA expression of *ERBB4* was determined for patients carrying c.1490-3C>T variant using reverse transcription quantitative polymerase chain reaction (RT-qPCR) with following primers: forward 5'-GAGTACTCTATAGTGGCCTG-3', reverse 5'-TTTGCCCCCTGTAAGCCATCT-3'.
